# Thyroid Carcinoma: A Review for 25 Years of Environmental Risk Factors Studies

**DOI:** 10.3390/cancers14246172

**Published:** 2022-12-14

**Authors:** Eva Kruger, Eman A. Toraih, Mohammad H. Hussein, Shaimaa A. Shehata, Amani Waheed, Manal S. Fawzy, Emad Kandil

**Affiliations:** 1 School of Medicine, Tulane University, New Orleans, LA 70112, USA; 2 Division of Endocrine and Oncologic Surgery, Department of Surgery, School of Medicine, Tulane University, New Orleans, LA 70112, USA; 3 Medical Genetics Unit, Department of Histology and Cell Biology, Faculty of Medicine, Suez Canal University, Ismailia 41522, Egypt; 4 Department of Forensic Medicine and Clinical Toxicology, Faculty of Medicine, Suez Canal University, Ismailia 41522, Egypt; 5 Department of Community Medicine, Faculty of Medicine, Suez Canal University, Ismailia 41522, Egypt; 6 Department of Medical Biochemistry and Molecular Biology, Faculty of Medicine, Suez Canal University, Ismailia 41522, Egypt; 7 Department of Biochemistry, Faculty of Medicine, Northern Border University, Arar 1321, Saudi Arabia

**Keywords:** thyroid, cancer, risk, environment, radiation, endocrine disrupting chemicals, volcanic

## Abstract

**Simple Summary:**

Increasing awareness of thyroid cancer-related environmental risk factors is considered an essential tool for cancer prevention through risk prevention/management. Various studies have identified correlations between environmental pollutants and thyroid cancer incidence rates, and others have proposed mechanisms for thyroid cancer development. This review seeks to consolidate the known environmental risk factors contributing to thyroid carcinoma to ensure that future research endeavors may identify key focus areas. These factors have been established as contributors to the development of thyroid carcinoma and thus require further investigation to establish mechanisms by which they act and influence thyroid pathology. Identifying pathophysiology involving these environmental risk factors can allow more rapid development of hazard reduction plans, exposure remedies, and medical treatments to prevent and perhaps even reverse disease course.

**Abstract:**

Environmental factors are established contributors to thyroid carcinomas. Due to their known ability to cause cancer, exposure to several organic and inorganic chemical toxicants and radiation from nuclear weapons, fallout, or medical radiation poses a threat to global public health. Halogenated substances like organochlorines and pesticides can interfere with thyroid function. Like phthalates and bisphenolates, polychlorinated biphenyls and their metabolites, along with polybrominated diethyl ethers, impact thyroid hormones biosynthesis, transport, binding to target organs, and impair thyroid function. A deeper understanding of environmental exposure is crucial for managing and preventing thyroid cancer. This review aims to investigate the relationship between environmental factors and the development of thyroid cancer.

## 1. Introduction

Environmental contaminants have been linked to several ailments, including metabolic disorders [1], allergies [2], diseases affecting male and female fertility [3], and malignancies [4], including thyroid carcinoma. Thyroid carcinoma is the most prevalent endocrine tumor [5], with enhanced diagnostic technology contributing to a recent rise in cases [6]. The highest incidence rates are found in both males and females in South Korea, while incidence rates are more significant for women than men in Eastern Asia, Australia/New Zealand, North America, Oceania, and Pacific Island nations [7]. Globally, thyroid cancer occurs predominantly in female patients compared to males, with approximately 2–4 times greater prevalence [8]. 

Thyroid carcinomas may originate from follicular thyroid cells or parafollicular thyroid cells. Follicular thyroid cell carcinomas progress through hypertrophy, hyperplasia, and benign (and, in some cases, eventually malignant) neoplasms. Papillary thyroid cancer (80–85%), follicular thyroid cancer (10–15%), poorly differentiated thyroid cancer (2%), and undifferentiated (anaplastic) thyroid cancer (2%) are the four subtypes of follicular cell-derived thyroid cancers [9]. These have an excellent prognosis, except for undifferentiated thyroid cancer [10].

**Figure 1:**
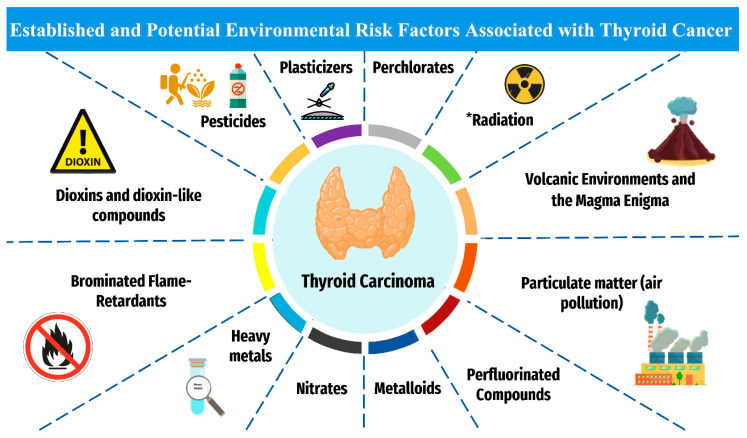
The established and plausible environmental risk factors for thyroid cancer. * Established risk factor for thyroid cancer. Otherwise, other factors are associated with potential increased risk for thyroid carcinoma.

Numerous intrinsic and extrinsic risk factors contribute to thyroid carcinogenesis. Intrinsic non-modifiable factors include biological sex, age, and familiar and hereditary conditions [11]. The ionizing radiation is considered as an established extrinsic factor, meanwhile other potential extrinsic factors are pesticides, persistent organic pollutants (POPs), endocrine-disrupting chemicals (ECDs), bisphenol A (BPA), phthalates, heavy metals, and polychlorinated biphenyls (PCBs) [12] (Figure 1).

Clinically relevant thyroid carcinoma risk trends positively with age; however, the malignancy risk of such nodules paradoxically declines with age [13]. Despite the significant prevalence of nodular thyroid disease worldwide—occurring in an estimated 4.2% of individuals aged 30–59—malignancy is notably rare, with a 4.5% occurrence [14]. According to the Italian AIRTUM (Associazione Italiana dei Registri Tumori, or Italian Associations of Cancer Registries) Working Group, there was a marked rise in the incidence rates of all malignant tumors among teenagers (15–19 years old) between 1991 and 2005, particularly Hodgkin’s lymphoma and thyroid cancer. The rise in cancer incidence that was seen through the 1990s has come to an end, except for thyroid cancer in young adults [15]. Genetic susceptibility and mutations play an essential role in the predisposition to thyroid cancer. Although there is a rise in the incidence of non-medullary thyroid cancer (NMTC), tumorigenesis of this type is not yet well-developed [16]. According to numerous studies, first-degree relatives of thyroid cancer patients are 8- to 12-fold more likely to develop thyroid cancer than the general population [17,18].

Many environmental factors have not yet been sufficiently studied to adequately deduce environmental pollutants’ impact on the human endocrine system, rendering prevention strategies for such pathology more challenging [19]. This review serves to comprehensively summarize the currently identified environmental toxicants and establish their relationship to thyroid carcinoma development.

## 2. Materials and Methods

The association between environmental risk exposure and thyroid cancer was explored by looking for publications and studies in international databases such as PubMed, Web of Science, and Scopus.

Environmental risk factors/exposure, thyroid, cancer, pesticides, persistent organic pollutants (POPs), including polychlorinated biphenyls (PCBs), endocrine disrupting chemicals (ECDs), bisphenol A (BPA), phthalates, heavy metals, and radiation were some of the keywords applied during the search.

## 3. Pollutants

**Figure 2:**
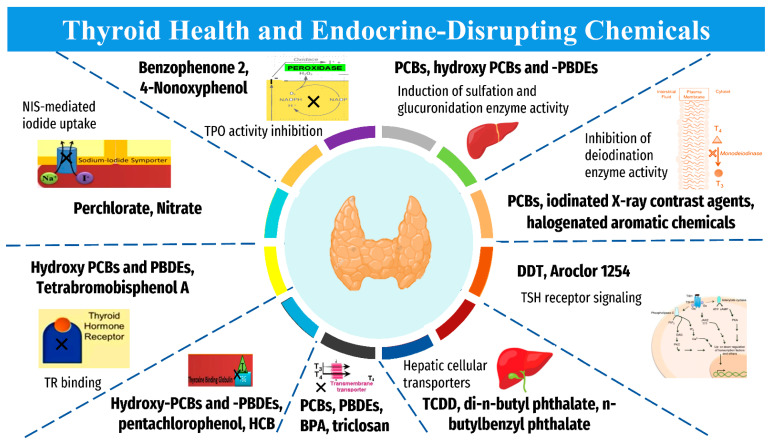
Mechanism of action of “Endocrine-Disrupting Chemicals” on thyroid homeostasis. “BPA, bisphenol A; DDT, dichlorodiphenyltrichloroethane; HCB, hexachlorobenzene; PCBs, polychlorinated biphenyls; PBDEs, polybrominated diphenyl ethers; TCDD, 2,3,7,8-tetrachlorodibenzo-p-dioxin; NIS, sodium iodide symporter; T3, triiodothyronine; T4, thyroxine; TR, thyroid hormone receptor; TBG, thyroid-binding globulin; TSH, thyroid stimulating hormone”. The collected data are based on Tang and colleagues [22].

Endocrine-disrupting chemicals (EDCs), including industrial chemicals and pesticides, are elements with deleterious health impacts [20]. Although they are understood to be detrimental to health and correlated with thyroid carcinoma, they do not necessarily decrease thyroid hormone levels, and current studies have been unable to reveal their exact mechanism of impact on human subjects [21]. See Table 1 for a consolidated summary of the established EDCs contributing to thyroid function disruption and/or potential increased risk of carcinoma and other pathology. The suggested mechanisms of action of the “EDCs” on thyroid are summarized in Figure 2. These chemicals can impact euthyroid state by several ways, including “disturbance of thyroid hormone biosynthesis through directly targeting the sodium-iodide symporter (NIS), interplaying with thyroid hormone transporters/receptors, interfering with the hypothalamic-pituitary-thyroid axis, and disrupting multiple molecular alterations associated with thyroid pathogenesis” [22]. These findings suggest that the exposure to these chemicals as modifiable risk factors should be considered, when investigating the potential increased incidence of thyroid cancer.

Human studies face a considerable challenge due to lifelong chronic exposure to various substances and the significant physiological diversity in thyroid hormone levels between individuals [49]. While global and national studies provide some insight into broad trends, specific dosing is difficult to determine for each pollutant, and several confounding variables from tandem chemical exposures interfere with and challenge exposome research [50].

### 3.1. Pesticides

Several studies have looked into the link between pesticides and thyroid cancer. The most extensively researched organochlorine pesticide (OCP) for its ability to behave as ECD is dichlorodiphenyltrichloroethane (DDT), along with its main metabolite dichlorodiphenyldichloroethylene (p,p-DDE). Thyroid hormone synthesis exhibits developmental flaws with DDT and HCB (hexachlorobenzene) [23]. Lindane, classified as a carcinogenic OCP based on “International Agency for Research on Cancer” (IARC) [51], was positively associated with thyroid carcinogenesis in a recent study [52]. Due to their toxicity and carcinogenicity, OCPs have been prohibited in many developed nations, yet they are utilized in many developed countries [53]. Atrazine (triazine herbicide) and Malathion (an organophosphate insecticide) were linked to increased thyroid cancer risk among individuals exposed to pesticides on farms [54]. Ref The improper use of OCP led to water and soil contamination with an accumulation of residues for decades [55]. The relationship between thyroid cancer risk and pesticide exposure has been investigated in numerous epidemiological studies. Zeng et al. found that there was a statistically significant link between thyroid cancer risk and occupational exposure to biocides and pesticides among workers in comparison with harmful exposure [56]. Ref In a prospective cohort study in Iowa and North Carolina, At enrollment (1993–1997) and follow-up (1999–2005), it found that pesticide usage involving fungicide (metalaxyl) and the OCP (lindane) was linked to increased hazard ratios (HR) thyroid cancer (HR = 2.03 and HR), respectively [52]. Exposure to Malathion, an organophosphate pesticide, in the environment has been linked to an increased risk of thyroid cancer in females who work and live in agricultural areas [57]. In Norwegian research, chlordane and HCB serum OCP metabolites have been positively linked to a rising incidence of thyroid cancer [58]. While HCB breaks down into very hazardous chemicals that target thyroid hormones, DDT reduces the function of thyroid hormones more directly. OCPs are EDCs whose chemical structure resembles triiodothyronine (T3) and thyroxine (T4). OCPs can competitively bind to thyroid transport proteins, interrupting thyroid hormone signaling and transport even at low doses [53]. Carbaryl is a non-persistent carbamate pesticide that disrupts thyroid hormone by antagonizing the receptor-signaling pathway, according to an in vitro study [59]. Malathion and parathion are widely used organophosphate pesticides used to control insects in agriculture, public health, and homes and has been linked to cancer in several different organs, but the evidence in human being remains scarce [60]. The carcinogenicity of Malathion and parathion is not yet well established. However, they induced thyroid follicular cell adenoma in male rats [61]. In an in vitro study, parathion induced genotoxicity via DNA and chromosomal damage in human cells, combined with inflammation, oxidative stress, receptor-mediated modulation, and apoptosis [62]. Although parathion and malathion usage has been restricted since the eighties, several developing countries still misuse them [63].

Furthermore, pesticides cause thyroid disruption mechanisms, including inhibiting iodine uptake, increasing clearance of thyroid hormones, interfering with iodothyronine deiodinase and thyroid peroxidase activities, interfering with cellular uptake of thyroid hormones, and modifying thyroid gene expression [64]. Consequently, alterations in thyroid hormone function could result in abnormal proliferation in the thyroid tissue, leading to oncogenesis. Multiple malignancies, including thyroid cancer, have been analyzed in various studies on other commonly used pesticides (alachlor, atrazine, chlorpyriphos, glyphosphate, imazethapyr, and metolachlor); however, they did not demonstrate a significant carcinogenic effect [23,24].

### 3.2. Plasticizers

Phthalates are frequently employed as plasticizers to increase materials’ malleability, flexibility, and plasticity. Phthalates can enter the human body through several methods, including ingestion, inhalation, and cutaneous exposure [27,65]. Many types of phthalates are still used in a variety of different industries, such as cosmetics, paints, food packaging, cleaning agents, and medical equipment, despite the current restriction on the use of some phthalates, such as Di-(2-Ethylhexyl) phthalate (DEHP), in children’s toys in the US and EU (tablet coatings, blood bags, tubes, drugs packaging, etc.). Phthalates do not bioaccumulate; instead, they are digested and primarily eliminated through the urine (within a few days or hours) [13,27]. The thyroid is one of the main target organs for phthalates’ capacity to disrupt the endocrine system [31]. Phthalates are particularly interesting EDCs due to continuous and long-term exposure by the entire world population. DEHP has been shown to bind to and activate the estrogen receptor (ER) and contribute to angiogenesis and tumor progression by controlling vascular endothelium growth factor (VEGF) [66]. This substance increases VEGF secretion in MELN cells (the Melbourne cells model) with continuous ER-alpha expression. Studies suggest DEHP increases the risk of developing differentiated thyroid cancer in patients with thyroid nodules [25], and DEHP metabolites in urine have been associated with papillary thyroid cancer [26]; however, establishing the precise mechanisms requires further study.

Another plasticizer that interferes with thyroid hormone is bisphenol A (BPA). BPA mimics natural hormones and interferes with their production and secretion in humans and other creatures, causing their endocrine systems to malfunction. BPA competes with endogenous estrogens to bind to membrane estrogen receptors and shares structural similarities with 17b-estradiol [29]. To date, there are few studies that have investigated the association between BPA and the risk of developing thyroid cancer in human [67]. However, according to some studies, BPA may affect thyroid function through several different mechanisms. BPA blocks the activity of human recombinant thyroid peroxidase (TPO) [68]. At the receptor level, BPA binds to the thyroid hormone receptor (TR) as a weak ligand and functions as an antagonist to T3, thereby preventing TR-mediated transcriptional activity [29,69]. In a recent study, Marotta et al. 2022 found that overweight/obese individuals subjects exposed to BPA showed high serum level of TSH [70]. This can sustain the hypothesis that BPA play role in TC development via thyroid hormones dysregulation, which in turn leads to TSH hyperstimulation and increased TC risk [70]. Fetuses and infants are especially susceptible to the consequences of BPA exposure, according to the current fetal genesis hypothesis of cancer [28].

### 3.3. Dioxins and Dioxin-like Compounds (Polychlorinated Biphenyls; PCBs)

Dioxin (2,3,7,8-tetrachlorodibenzo-p-dioxin; TCDD) is one of the most toxic persistent organic pollutants (POPs) that are created from uncontrolled waste combustion in the presence of chlorine [71]. Dioxins are a group of chemical compounds consisting of 75 polychlorinated dibenzo-p-dioxin (PCDD) and 135 polychlorinated dibenzofurans (PCDFs) [72]. Dioxin bioaccumulates in the food chain, particularly in fat, with elimination half-lives ranging from 3 to 10 years [73]. Animals experiment and human research proved that TCDD is a potent carcinogen that can alter numerous endocrine pathways [74]. A recent study measuring PCDD/DFs using serum from a general population found that PCDD/DFs had a significant positive association with thyroid cancer development [74]. Another animal study found that TCDD exposure controls the script of an endothelial carcinogen network that leads to thyroid carcinoma [75]. Dioxins dysregulate thyroid hormone and could induce thyroid cancer through several mechanisms as binding to the protein transport of thyroid hormones [76], direct damage to the thyroid tissue, and the activation of thyroid-metabolizing enzymes [77].

The worldwide market for electronic products has been continuously growing. Improper e-waste disposal will inevitably release polychlorinated biphenyls (PCBs) and other toxicants into the environment [78]. The most common uses of PCBs include flame-retardant coatings, lubricants, elastic sealants, paints, and industrial products such as dielectric fluids in transformers and capacitors [79]. Their production was banned in most countries by the 1980s due to environmental persistence. Before their production was outlawed in the late 1970s, (PCBs) were a class of synthetic, lipophilic POPs widely utilized in industrial and consumer products for decades. 

Due to PCBs’ lipophilicity, which causes them to bioaccumulate in the food chain, the consumption of large fish (such as tuna, shark, and swordfish) is the leading cause of human exposure. PCBs can cause thyroid disruption [27]. Notably, the PCBs metabolites, including sulfated, hydroxylated, and methyl sulfones, are more toxic than PCBs [80]. Although the carcinogenicity of PCBs on thyroid tissue is not well understood, a few occupational studies show an increased risk of thyroid cancer in occupational settings, such as a rise in thyroid tumor mortality rate among capacitor manufacturing workers [81]. In a recent epidemiologic study, Zhuo et al., 2022 found that specific PCB exposure (PCB-74, PCB-99, PCB-105, PCB-118) was associated with an increased risk of papillary thyroid carcinoma, particularly type PCB-118. High levels of serum PCBs were detected by gas chromatography in samples collected nine years before papillary thyroid carcinoma diagnosis among US military service members [82]. A toxicological animal study observed that PCB-118 caused a reduction in serum T4 levels in a dose-dependent manner and induced thyroid gland lesions liability in female rats [83]. According to the literature, PCBs are structurally similar to thyroid hormone and may influence thyroid function through receptor-mediated mechanisms or alter thyroid hormone metabolism [84].

Additionally, PCBs stimulate the proliferation of cancer cells through the imbalance of molecular and signal pathways [85]. Based on a recent molecular docking study, it is revealed that PCBs can dysregulate thyroid hormone by interfering with PIK3R1, RXRA, MAPK1, MAPK3, and PI3K-Akt signaling pathways. The previous intersection genes play the leading role in thyroid cancer genesis [85].

### 3.4. Perfluorinated Compounds

Perfluorinated compounds (PFC) have surface protective qualities and include substances like perfluorooctanoic acid (PFOA) and perfluorooctane sulfonate (PFOS). PFCs are a diverse class of substances with a wide range of uses, including as additives in paper goods, stain-repellents for textiles, and in aqueous film-forming foams used to prevent electrical fires. They have been added to the Stockholm list [34] due to their stain- and oil-resistant PFC properties, which are employed in numerous consumer products and are quite persistent in the environment. An extensive investigation involving 506 workers at a PFC manufacturing plant revealed a negative correlation between PFOA and FT4, suggesting that exposure to significant PFOS may impair human thyroid function. Results of the more recent NHANES study conducted in the US revealed that men and women with high levels of PFOA and PFOS are more likely to report having thyroid disease [34]. PFC substances seem to affect how thyroid hormones are metabolized.

### 3.5. Brominated Flame-Retardants

Brominated flame retardants (BFRs) are frequently utilized in consumer goods such as electronics, cars, plastics, and textiles to reduce flammability [31]. Polybrominated diphenyl ethers (PBDEs) have been widely utilized as flame retardants in commercial and domestic products in the United States since the 1970s, including foam padding, textiles, electronics, cars, and airplanes [28,31]. PentaBDE, OctaBDE, and DecaBDE were used in the United States until 2004. The production of PentaBDE and OctaBDE was prohibited due to worries about toxicity and the bioaccumulation of PBDEs in blood and breast milk. The US Environmental Protection Agency published regulations in 2010 to further restrict the importation of goods containing these substances. Only one of the congeners, decabromodiphenyl ether (BDE-209), the primary congener in the DecaBDE formulation, has been studied in humans, thanks to Aschebrook-Kilfoy and colleagues [86]. The small number of cases does not imply a higher risk of thyroid cancer despite the apparent disruption of thyroid homeostasis caused by PBDEs in the environment. Kim et al. examined 36 epidemiological studies on the potential effects of BFR intake, such as impacts and changes on thyroid function [87]. The health effects of exposure to BFRs are well documented, but more epidemiological research is required, especially among children [12].

### 3.6. Perchlorates

Because of its well-known antithyroid properties, perchlorate has been used to diagnose and treat thyrotoxicosis. It is utilized in manufacturing weapons and pyrotechnics, and in previous years, the US drinking water supply’s perchlorate content raised concerns. The most significant pathways of exposure for the general public are through the consumption of food and water that contain perchlorates [32]. Despite the well-documented antithyroid effects of perchlorate, it is still unclear what the lowest practical level of environmental pollution is. Given the inconsistent findings of human observational research, it is still unclear whether naturally occurring quantities of perchlorate ions impact human thyroid function, particularly in neonates.

### 3.7. Heavy Metals

Heavy metals are substantial “environmental pollutants”, and their toxicity is a challenge of increasing significance for “evolutionary, ecological, nutritional, and environmental” causes [88]. Health effects from metals in food, water, and air have been reported [28,38,39,40,41,42,43,44,45]. While some metals, such as chromium, iron, copper, magnesium, and zinc, are necessary for life, they are toxic when they exceed their threshold limits. The toxic impacts of other metals, such as aluminum, nickel, cadmium, mercury, and lead, even though these substances do not have any biological role, are detrimental to human health [88]. Heavy metals include waste products from incinerators, combustion of gasoline or diesel fuel as essential components of particulate matter with a diameter of 10 microns or less (PM10) and fine particles that are 2.5 microns or less in diameter (PM2.5) produced from cars, trucks, and airplanes. Also include smelters, paints, insecticides, and agricultural products like disinfectants which can be absorbed through inhalation, ingestion, or even skin contact [28].

Arsenic, beryllium, cadmium, chromium, and nickel are identified as either definite or probable carcinogens according to IARC [89]. In particular, cadmium, mercury, arsenic, lead, manganese, and zinc exhibit EDCs’ capacity to disturb the hormonal system as well as they can act as carcinogens, promoting malignant transformation [35].

Cadmium is a category I carcinogen that can build up in the thyroid, liver, pancreas, and kidneys. Cadmium levels in the thyroid are correlated with blood levels. Multinodular goiter, thyroglobulin hyposecretion, and parafollicular cell hyperplasia are common in chronic cadmium exposure [30]. Cadmium can interfere with thyroid gland function even at extremely low ambient exposures, both at the target gland and extra gland level, according to several epidemiological and experimental studies [90,91,92,93,94]. Also, it has been established that cadmium contributes to thyroid cancer and autoimmune diseases [92,95]. One central mechanism that has been suggested behind the Cadmium effect on the thyroid gland is the oxidative stress and upregulation of multiple apoptotic players, including “nuclear condensation, DNA fragmentation, Bax integration, cytochrome c release”, etc., as concluded by Buha and colleagues in their review [92]. 

High amounts of cadmium in thyroid tissue have been linked to more advanced thyroid cancer stages in women, according to a Korean study. This finding is of particular significance given that traditional Korean cuisine is linked to high levels of cadmium exposure [35].

Enzymes with manganese (Mn) support several metabolic pathways [96]. Manganese exposure to the general public is primarily derived from diet, though in some areas drinking water may also be a source [97]. It has been shown that abnormal thyroid hormone levels caused by high Mn exposure during pregnancy are associated with poor neurodevelopmental consequences [98]. However, a measurable rise in thyroid cancer incidence due to exposure of mice to Mn was not evident, and the available epidemiological and occupational evidence is still inconclusive [37].

Lead (Pb) has generated debate surrounding its carcinogenicity. Li and colleagues studied 96 papillary thyroid carcinomas (PTCs), 10 nodular goiters, and seven thyroid adenomas in Chinese patients with various thyroid conditions to determine the relationship between Pb and thyroid function. Serum triiodothyronine (T3), free triiodothyronine (FT3), free thyroxin (FT4), thyroid-stimulating hormone (TSH), and serum lead levels were assessed, and lead levels were substantially greater in thyroid adenoma and decreased in nodular goiter when compared to PTC (*p* < 0.05). Even after sex stratification, the disparity increases among women. In the PTC group, there was a negative correlation between serum lead and TSH (rs = 0.27, *p* < 0.05). In the PTC group, T3 was quartile-wise positively correlated with lead (rs = 0.61, *p* < 0.05). None of the groups showed any measurable relationships between lead and FT3 or FT4. According to the study, lead may play various etiological roles in thyroid conditions, including thyroid cancer [36].

The most prevalent form of the metal vanadium (V), which can exist in many oxidation states, is vanadium pentoxide (V2O5). Vanadium compounds are all poisonous [99]. Some authors have linked vanadium to thyroid cancer; however, no research has examined the possibility of developing thyroid illness in people or animals who have been exposed to vanadium [12,100]. In their investigation, Fallahi et al. assessed how V2O5 affected the proliferation/chemokine release of healthy thyrocytes. They found that V2O5 can induce T-helper 1 chemokines secretion (e.g., interferon and tumor necrosis factor) and enhance the impact of these chemokines in the thyroid [38].

### 3.8. Metalloids

Thyroid hormone synthesis and function depend on micronutrients, primarily iodine (I) and selenium (Se) [39]. Iodine and selenium have been demonstrated to impact thyroid autoimmunity [27] significantly. Iodine prophylaxis lessens thyroid diseases caused by iodine insufficiency [101].

During a modest iodine shortage, a 53% greater prevalence of spontaneous overt hypothyroidism (likely autoimmune) was seen in the Danish population. On the other hand, too much iodine is linked to the development of autoimmune thyroiditis [40]. For further information, see Section 4.1. Radioiodine ^131^I is used in medical diagnostic and treatments for differentiated thyroid cancer (DTC) to remove any remaining normal thyroid tissue after thyroidectomy.

In a healthy state with a regular metabolism, selenium, a trace mineral vital to human health, performs several tasks as selenoprotein [41]. The effects of selenium, whose daily requirement is between 60 and 75 g, include those on immune responses, homeostasis, cell development, and antiviral defense. Because of its role in various enzyme activities, including glutathione peroxidases, deiodinases, and thioredoxin reductases, which actively contribute to the defense against free radicals and oxidative damage, it is required for optimal thyroid function in humans [42]. Although its significance in cancer treatment is still debatable, selenium plays a role in developing thyroid carcinoma (TC) [42]. The endocrine system, especially thyroid function, may be negatively impacted by a high selenium intake [40]. A Chinese cross-sectional study in two counties of Shaanxi Province examined the connections between dietary variables, pathological thyroid diseases, and selenium levels. The prevalence of pathological thyroid disorders (subclinical hypothyroidism, hypothyroidism, AT, and enlarged thyroid) in the adequate-selenium concentration group was considerably lower than that in the low-selenium group (18.0 versus 30.5 percent; *p* < 0.001) [43]. Reduced odds ratios (95% confidence interval) of subclinical and clinical hypothyroidism, autoimmune thyroiditis (AT), and enlarged thyroid were found to be linked with elevated circulating selenium levels. With the help of measurements of thyroid-stimulating hormone (TSH), thyroid hormones, TPOAb and thyroglobulin antibodies (TgAb) levels, thyroid echogenicity, and thyroid hormone levels after six months of l-selenomethionine treatment, Wu et al. evaluated the true efficacy of selenium supplementation in Hashimoto’s thyroiditis [43]. They found that short-term l-selenomethionine supplementation has a limited effect on euthyroid HT’s natural course.

### 3.9. Nitrates

Nitrates are widespread pollutants. Nitric oxide (NO), a recognized carcinogen that is implicated in a variety of pathogenic pathways, is produced in excess when high levels of nitrates are absorbed orally. This results in hypoxia, especially in children and adolescents.

Nitrate contamination threatens thyroid functions because it can prevent the thyroid from absorbing iodide from food and drinking water [44]. Thyroid-stimulating hormone (TSH), a sensitive indicator of thyroid function, is increased as a result of compensatory thyroid hormone production being reduced by nitrate. However, a cohort study of postmenopausal women in Iowa did not find that exposure to high nitrates in the public water supply correlated to high levels of thyroid dysfunction [44]. A high TSH release has been shown to induce hypertrophy and thyroid disease, including carcinoma, in animals [102].

Consuming foods high in nitrates was not substantially linked to an increased risk of developing gastric cancer, according to a meta-analysis by Xie et al. (RR = 1.24, 95% CI = 0.89–1.72); however, people who consume more nitrites have a higher chance of developing thyroid cancer (RR = 1.52, 95% CI = 1.12–2.05) [103]. Inoue-Choi et al. found that the risk of developing thyroid cancer was 2.6 times higher in women with >10 years of exposure to public water supplies with levels surpassing 5 mg/L NO3-N than in women whose drinking water had never surpassed this level [45].

When thyroid-stimulating hormone levels were measured in an Amish community in Pennsylvania (USA), nitrate concentrations in private wells were linked to an increased prevalence of subclinical hypothyroidism in women but not men [44]. Additionally, therapeutic irradiation (^131^I) raises NO levels in salivary gland tissue. Numerous studies have demonstrated that NO is an intrinsic radiosensitizer; in fact, administering an inhibitor of NO production can improve the function of radio-exposed salivary glands. NO-dependent effects include genomic instability and accumulation of DNA replication errors. These effects may be seen in unirradiated cells due to signals from neighboring radiated cells. NO may quickly diffuse through the cytoplasm and plasma membranes due to its hydrophobic qualities, dissipating radiation from irradiated cells to nearby cells without the need for gap-junctional intercellular communication [46].

### 3.10. Air Pollution

Urban air pollution is a widely acknowledged cancer risk factor, according to the IARC of the World Health Organization (WHO), which oversaw a modification to the classification of cancer-causing chemicals [47]. The “sum of all solid and liquid particles of organic and inorganic materials suspended in air”, of which many of them are harmful, is referred to as particulate matter (PM) [104]. More people are affected by PM than other pollution [105]. Sulfate, nitrates, ammonia, sodium chloride, black carbon, mineral particles, and water vapor are the main components of PM [106]. PM10 particles, which are 10 microns in diameter or smaller, can enter the lungs and become lodged there. While particles with a diameter of 2.5 microns or smaller (PM2.5) can cross the lung barrier and enter the blood, they are far more harmful to human health. Chronic exposure to such particles increases the risk of lung cancer, as well as respiratory and cardiovascular problems [107].

According to the IARC’s 2015 position statement, almost 80% of European inhabitants are exposed to high quantities of tiny particles (PM2.5 and PM10) that have been linked to human cancer [33]. A significant prospective study found that outdoor air pollution (PM2.5, NO2, and O3) was positively linked with colorectal, kidney, and bladder cancer death [47]. In a retrospective population-based study conducted in Shanghai, China, Cong studied the impact of outdoor air pollution from waste gas emissions on cancer occurrences. More than 550,000 new cancer patients were recruited and assessed. Cancer incidence of various types, including TC, was found to have a statistically significant positive association with pollution caused by waste gas emissions [48].

## 4. Radiation

The association between radiation exposure and the occurrence of TC has been well documented [108,109,110,111]. The incidence and causes of death from thyroid cancer in the Life Span Study (LSS) sample in Nagasaki and Hiroshima have been investigated [112,113]. It has been established that the rise in TC is caused by increasing radiation dose and exposure time [114,115,116]. Importantly, medical radiation such as radiotherapy could increase the risk of secondary thyroid cancer in some patients, particularly children who have received radiotherapy to treat leukemia, Hodgkin lymphoma, and neuroblastoma [117]. 

The thyroid tissue is sensitive to radiation. Ionizing radiation proved to be carcinogenic (group 1) by IARC [118]. Its ability to deliver high amounts of energy to cells causes direct DNA distraction [119]. Recent research stated that exposure to diagnostic radiography as X-ray was linked with an increased risk of thyroid cancer, particularly thyroid microcarcinomas [120]. Additionally, childhood exposure to computerized tomography (CT) scanning was associated with a 40% increase in thyroid cancer [121]. The effects of non-ionizing radiation (NIR) on the thyroid have not yet been well developed [119]. The wide use of cell phones raises alarming concerns regarding potential harmful effects. However, some research studied the association between cell phones and thyroid cancer with conflicting outcomes [119,122,123,124]. Long-term and frequent cell phone use carries potential risks for thyroid cancer, especially among females [119]. Low-frequency electromagnetic radiation has sufficient energy to heat and vibrate molecules. Importantly, NIR can lead to oxidative stress in human cells, damaging DNA [125]. 

### 4.1. Anthropogenic Radiation

The main fission product of uranium and plutonium is iodine-131 (^131^I), which accounts for 3% of all fission products. It is connected to nuclear energy and diagnostic and therapeutic processes in medicine. It contributed significantly to the health risks associated with the Chernobyl disaster, open-air atomic bomb testing in the 1950s, and the initial weeks of contamination risk following the Fukushima nuclear crisis [126]. From a therapeutic perspective, radioiodine ^131^I is given to patients with differentiated thyroid cancer (DTC) to remove any remaining normal thyroid tissue following thyroid surgery, treat any remaining microscopic disease (adjuvant treatment), and treat macroscopic or metastatic illness [127].

The effectiveness of ^131^I therapy depends on the tumoral thyroid tissue’s capacity to absorb iodine from the blood via the sodium/iodine transporter membrane [128]. Because tumoral tissue expresses this transporter less than healthy tissue, there may be less uptake of ^131^I. However, the primary goal of treatment is cell death, which is achieved through the creation of oxidative species like free radicals at the intracellular level, DNA damage, and beta radiation from ^131^I [128]. As expert recommendations are mainly based on data interpretation from observational retrospective research, the use of ^131^I to treat well-differentiated thyroid cancer is still a controversial topic [129]. According to the previous recommendations of the “American and British Thyroid Associations”, the “European and American Societies of Nuclear Medicine”, the “European Consensus Group”, and the “National Comprehensive Cancer Network”, the use of ^131^I was justified in high-risk, intermediate-risk, and low-risk thyroid carcinoma [127]. However, recent results of a prospective trial concluded that “a follow-up strategy that did not involve the use of radioiodine was non-inferior to an ablation strategy with radioiodine regarding the occurrence of functional, structural, and biologic events at three years” in patients with low-risk thyroid carcinoma subjected to thyroidectomy [130].

Childhood exposure to radioisotope ^131^I was linked to a higher risk of thyroid cancer [131]. Iodine supplementation and iodine deficiency both alter this risk. So, if radioactive iodine results from radiation accidents or during medical diagnostic and therapeutic procedures, regular iodine supplementation in iodine-deficient populations may significantly lower the risk [101]. Even though thyroid tumors can be brought on by other ionizing radiation, such as the alpha particles from plutonium-238 (^238^Pu) [132], the thyroid gland is susceptible to radioiodine exposure.

Children in Japan were exposed to radiation from US atomic bombings in 1945, and teenagers, in particular, exhibited an elevated risk of acquiring thyroid cancer five decades later [112,133]. The Chernobyl disaster in 1986 resulted in the most uncontrolled radioactive discharge into the environment ever seen in a civilian operation. Radiation in Belarus and Ukraine was significantly increased by ^131^I [134,135]. High thyroid levels, particularly in younger people, were caused by 131I contamination of fresh milk and the lack of rapid countermeasures [112]. The prevalence of thyroid peroxidase antibodies was higher in radiation-exposed children and adolescents exposed to radioactive fallout 13–15 years after the Chernobyl accident (6.4% versus 2.4% in unexposed children). The evidence of danger among exposed adults is murky since no higher risk was discovered among adult Chernobyl liquidators [126]. In Chernobyl, there was an increased incidence of thyroid cancer among exposed children [112]. Hence, thyroid cancer incidence was significantly lower in children born after the Chernobyl disaster [136]. More than 11,000 thyroid cancer cases were exposed during childhood in Ukraine, Belarus, and Russia [137]. Importantly, PTC was the most thyroid cancer type among these children [138,139]. Molecular analyses in early childhood thyroid cancer cases revealed an extremely high prevalence of genome rearrangements between the Rearranged During Transfection (*RET*) gene and the *PTC3* gene (*RET*/*PTC3* rearrangement) located on the same chromosome 10 [140,141]. *RET*/*PTC* oncogenic rearrangements are now identified as principal triggering mutations in radiation-related and sporadic childhood PTC [135,142]. The later event of the nuclear catastrophe at the Tokyo Electric Power Company-operated Fukushima Daiichi Nuclear Power Station, which took place in March 2011, was described by Nagataki and Takamura as having radiation effects on the thyroid. The incident discharged 120 Peta Becquerel of radioiodine into the environment, much lower than the Chernobyl disaster. Most children, including those under one year old, had thyroid radiation doses of less than 100 mSv (intervention threshold for continuous iodine delivery) since residents close to the Fukushima nuclear facility were evacuated within a short time. Of the affected children, over 280,000 have been screened, and 90 have been diagnosed with thyroid cancer to date (approximate incidence: 313 per million) [143]. Experts from Japan and international organizations, including the WHO, United Nations Scientific Committee on the Effects of Atomic Radiation (UNSCEAR), International Commission on Radiological Protection (ICRP), International Atomic Energy Agency (IAEA), and International Agency for Research on Cancer (IARC), reviewed and examined the thyroid cancer findings in Fukushima, emphasizing that the high detection rate of thyroid cancer and benign abnormalities resulted in the higher incidence rate. However, no incidences of thyroid cancer were found in the most vulnerable population, very young children, indicating that the increase is more likely reflective of high screening rates than radiation exposure [144].

A recent meta-analysis revealed that research has not linked living close to nuclear facilities to an increased risk of thyroid cancer [143]; however, because there was significant variation among incidence studies, care must be used when interpreting the findings. Modern nuclear power stations’ radioactive releases are probably too small to be statistically detectable.

### 4.2. Natural Radiation

The radioactive decay of uranium, which is present in rocks and soil, results in the production of radon (Rn), an odorless, tasteless, and colorless natural gas. A component of uranium-238′s (^238^U) radioactive decay chain is radon-222 (^222^Rn). The decay cycle of thorium-232 (^232^Th) produces radon-220 (^220^Rn). Radon naturally decays into short-lived radioactive isotopes humans can breathe and drink [143]. Springs and wells that draw groundwater serve as drinking water sources in many nations [145]. Compared to surface water from reservoirs, rivers, or lakes, these water sources typically have greater radon concentrations [143]. Because radon has been linked to lung cancer, exposure to levels above 4 picocuries per liter (pCi/L) is risky. After tobacco smoke, radon is the second-leading cause of lung cancer worldwide [143]. Radon is classified as a category I carcinogen. The volcanic region of Catania Province, Sicily (Italy), has been found to have a high Rn concentration, and its population is reported to have a higher incidence of thyroid cancer than others of non-volcanic, adjacent regions. However, the authors of this study were unable to draw any conclusions about the relationship between Rn exposure and the risk of thyroid cancer [146]. An ecological study conducted in three US States (Iowa, New Jersey, and Wisconsin) revealed no connection between elevated radon levels and the prevalence of thyroid cancer in women. In fact, from 1990 to 2013, New Jersey (N = 16,906), Wisconsin (N = 7250), and Iowa (N = 4236) had the highest number of female thyroid cases documented. For each of the three states, Pearson correlation coefficients were determined between radon levels over 4 pCi/L and the age-adjusted thyroid cancer incidence rate, although none of the comparisons revealed a connection [147]. Additionally, a different Pennsylvania study found no connection between cumulative radon levels and the occurrence of thyroid cancer [148]. 

## 5. Volcanic Environments and the Magma Enigma

In 1981, an increased incidence of thyroid cancer in environments with active volcanoes came to light. Iceland and the Philippines have been noted to have higher thyroid cancer rates than otherwise similar, non-volcanic environments [149]. A 30-year retrospective study on the incidence of thyroid cancer in Iceland, which covered the years 1955–1984 and included 406 registered cases, found that the incidence was 9.5/100,000 for females and 3.4/100,000 for males, twice as high as in other Nordic regions. Thyroid cancer was similarly found in 69.4% of 72 Filipino patients with thyroidectomies due to nodular goiter, compared to 38.9% of 72 controls matched for various variables. This finding highlights the elevated risk for thyroid cancer in Filipino people. Compared to White women born in the US, the rate of TC in Filipino women born in the Philippines was 3.2 times greater. Significantly, Filipino women born in the US did not face higher risks [150].

In areas around volcanic sites, non-anthropogenic pollution of soil, water, and the environment with heavy metals has been reported, including in Sicily, Italy, which sees regular eruptions of Mt. Etna [149]. Humans in these areas have also been contaminated by non-anthropogenic pollution with heavy metals through the food chain, as shown by statistically significantly higher metal levels in urine and scalp hair than in nearby non-volcanic areas. In addition, sulfur competes with molecules containing selenium for uptake by plants, reducing selenium availability. The substantial soil acidification that occurs after volcanic eruptions, as was observed, for instance, during the eruption of Etna in 1991, has long-term adverse effects on the body, altering the redox state, impairing defense mechanisms, causing inflammation, and accelerating cancer. DNA damage is more common in people who live in volcanically active areas than in people who live in nearby control areas [151]. The hormesis effect, a biphasic dose-response relationship observed in vitro for many metals, including arsenic, cadmium, copper, and mercury provides some insight into the precise impact of certain metals at various concentrations in various human tissues. The biological effects of this phenomenon are stimulated at low doses (µM or even nM) and inhibited at more significant quantities. The exact mechanism of hormesis is unknown, but it is known that chronic exposure to various toxicants at low levels can have significant toxicological effects [152].

## 6. Teratogens and Developmental Hazards

Proper control of environmental pollution could have a positive impact on population health. A novel theory posits that fetal exposure to EDCs during pregnancy may lead to endocrine dysfunction later in life [153,154]. EDCs have been found in amniotic fluid [155], umbilical cord blood [29], and other body fluids [156]. Following birth, EDCs exposure could be maintained through breastfeeding [157,158], infant meals [159,160], and direct environmental interaction. Plentiful manufactured sources of EDCs lead to an estimated human exposure of 1.7–52.1 g/kg/day to phthalates, for example, and under some conditions, newborns can be exposed to up to three times as much [155].

EDCs contribute to the development of cancers through both genetic and epigenetic processes. There is growing evidence that EDCs like PCBs, BPA, and phthalates can affect the thyroid in humans. According to a Yoshinaga study, environmental exposure to hydroxylated PCBs during the first trimester of pregnancy may impact the newborn’s thyroid hormones [161]. It has been demonstrated that early exposure to some environmental chemicals with endocrine-disrupting activities, such as pesticides, may affect a newborn’s thyroid hormone state, increasing the likelihood of developing a thyroid tumor in adulthood [162].

Most oncogenic fusions detected in cancer are caused by chromosomal rearrangements brought on by medicinal and pharmacological factors. Regions with deletions and chromosomal rearrangements have been found to include weak DNA spots, which are vulnerable to several toxins [163].

Thyroid cancer is thought to be influenced by epigenetic processes, particularly abnormal DNA and microRNA methylation [164]. The post-translational alteration of histone proteins (via acetylation, methylation, phosphorylation, ubiquitylation, sumoylation, proline isomerization, and adenosine diphosphate-ribosylation) is another well-studied epigenetic change that affects gene expression [165,166]. The disruption of genetic material caused by rearrangements may result in abnormalities, the development of oncogenic fusion proteins, or gene silencing, frequently cited as the primary method of tumor suppression [165].

Point mutations in the BRAF gene (which accounts for 40% of PTC cases), the RAS gene (15% of PTC cases, primarily the follicular form), or the RET/PTC (REarranged during Transfection-RET) rearrangement are only a few examples of the genetic variables that contribute to the development of PTC (18% of PTC cases).

Some environmental factors, such as benzene, found in cigarette smoke/car exhaust, diethylnitrosamine, present in pesticides, cigarette smoke, cured meat, and cancer chemotherapy, can significantly increase “fragile site breakages” with subsequent increase in the thyroid cancer risk [149,167].

## 7. Conclusions

This review explored how environmental risk factors may lead to an increased risk of thyroid cancer. Experimental studies have shown that several chemical groups may adversely disrupt thyroid function; however, only the impacts of environmental PCB levels have been thoroughly researched in epidemiological studies. For most substances, the association of exposure with the risk of thyroid cancer has not been thoroughly investigated, and the findings are not always consistent. There is strong evidence that PCBs impair thyroid function, and research from other halogenated substances, BPA, certain metals, metalloids, and phthalates suggests that these chemicals may also disrupt thyroid function and thus poses a risk for tumor promotion.

Based on the published data, we can hypothesize that thyroid cancer may result from the simultaneous coexistence of several conditions, including the genetic background of the individuals, environmental risk factors exposure, and nutritional factors, among others. Excessive nitrate uptake from drinking water increases nitrite production, causing hypoxia, especially in children, and from an excess of NO, a carcinogenic compound. Salivary glands are exposed to radiation via natural and therapeutic means, such as by dental X-ray examination. Hazardous geographic environments, such as volcanic zones and areas exposed to air pollution, lead to a lack of selenium or iodine. If any of these processes coincide with thyroid radiation exposure, markedly elevated NO concentrations in the body enhance the carcinogenic effect of radiation.

Unavoidable lifetime human exposure to a combination of these environmental toxins raises serious questions about their ability to cause thyroid cancer, mainly if exposure occurs during delicate developing stages. The development and severity of thyroid cancer in children and adults may be determined partly by fetal exposure to environmental effects during pregnancy. Further research should also focus on preventing overdiagnosis and overtreatment, both of which pose potential health concerns to individuals, along with excessive financial burden for patients in some countries.

Future studies should focus on determining safe levels of exposure to persistent organic pollutants following rigorous protocols as recommended by Zhang et al. [168]. This includes accounting for potential confounders, such as occupation, lifestyle, or exposure to several substances that may increase the risk of thyroid cancer. More rigorous evidence-based research and an integrated interdisciplinary approach, including “improved analytical tools and exposome databases, along with individual exposure measurements and effect-directed analysis”, according to Tang et al. [22], are required to understand better the mechanisms that lead to thyroid cancer and provide a complete picture of actual environmental exposures, and ultimately, to discover the specific environmental factors that increase the chance of disease occurrence in humans.

## Figures and Tables

**Table 1:** Summary of endocrine-disrupting chemicals and their impacts on thyroid health.

EDCs	Examples Discussed	Impact on Thyroid Health	Sources
Pesticides	DDT, HCB	Impairment of thyroid hormone development	[23,24]
Plasticizers	DEHP, BPA	Enhanced risk of papillary thyroid cancer, receptor agonist mimicry (TPO, TR, estradiol)	[7,25,26,27,28,29]
PCBs		Thyroid disruption	[27]
PFCs	PFOA, PFOS	Decreased T4 level/impaired thyroid function	[30]
BFRs	PBDEs	Changes in thyroid function	[28,31]
Perchlorates		Changes in thyroid function	[32]
Metals	Cd, Pb, Mn, V	Multinodular goiters, thyroglobulin hyposecretion, parafollicular cell hyperplasia (Cd), increased risk of thyroid adenoma (Pb), abnormal thyroid hormone levels (Mn), thyroid inflammation and cancer correlation (V)	[28,30,33,34,35,36,37,38,39,40,41,42,43,44,45]
Metalloids	Se	Autoimmune thyroiditis and enlarged thyroid	[28,39,40,42,43,45]
Nitrates		Thyroid cancer risk possibly increased	[44,45,46]
Air Pollution	Particulate matter	Thyroid cancer risk possibly increased	[33,47,48]

DDT: dichlorodiphenyltrichloroethane, HCB: hexachlorobenzene, DEHP: Di-(2-Ethylhexyl) phthalate, BPA: bisphenol A, TPO: thyroid peroxidase, TR: thyroid hormone receptor, PCB: polychlorinated biphenyls, PFCs: Perfluorinated compounds, PFOA: perfluorooctanoic acid, PFOS: perfluorooctane sulfonate, T4: thyroxine, BFRs: Brominated flame retardants, PBDEs: polybrominated diphenyl ethers; Cd: cadmium, Pb: lead, Mn: manganese, V: vanadium, Se: selenium.
